# Coronavirus Disease-2019 Conundrum: RAS Blockade and Geriatric-Associated Neuropsychiatric Disorders

**DOI:** 10.3389/fmed.2020.00515

**Published:** 2020-08-11

**Authors:** Aline Silva de Miranda, Antonio Lucio Teixeira

**Affiliations:** ^1^Laboratório Interdisciplinar de Investigação Médica, Faculdade de Medicina, Universidade Federal de Minas Gerais, Belo Horizonte, Brazil; ^2^Laboratório de Neurobiologia, Departamento de Morfologia, Instituto de Ciências Biológicas, Universidade Federal de Minas Gerais, Belo Horizonte, Brazil; ^3^Instituto de Ensino e Pesquisa Santa Casa BH, Belo Horizonte, Brazil; ^4^Neuropsychiatry Program, Department of Psychiatry and Behavioral Sciences, McGovern Medical School, University of Texas Health Science Center at Houston, Houston, TX, United States

**Keywords:** COVID-19, SARS-CoV-2, RAS, ACE2, ACEIs, ARBs, geriatrics, neuropsychiatric disorders

## Abstract

Coronavirus Disease 2019 (COVID-19) is caused by the novel severe acute respiratory syndrome coronavirus 2 (SARS-CoV-2), which primarily targets the human respiratory system and may lead to severe pneumonia and ultimately death. Mortality rate is particurlarly high among people beyond the sixth decade of life with cardiovascular and metabolic diseases. The discovery that the SARS-CoV-2 uses the renin-angiotensin system (RAS) component ACE2 as a receptor to invade host epithelial cells and cause organs damage resulted in a debate regarding the role of ACE inhibitors (ACEIs) and angiotensin receptor blockers (ARBs) therapies during COVID-19 pandemic. Some authors proposed the discontinuation of ACEIs and ARBs for cardiovascular, kidney, and metabolic diseases, while expert opinions have discouraged that due to limited empirical evidence of their negative effect on COVID-19 outcomes, and that withdrawing treatment may contribute to clinical decompensation in high-risk patients. Moreover, as cardiovascular and metabolic diseases are associated with neurodegenerative and psychiatric disorders, especially among older adults, a critical appraisal of the potential positive effects of ACEIs and ARBs is highly needed. Herein, we aim to discuss the conundrum of ACEIs and ARBs use in high-risk patients for COVID-19, and their potential protective role on the development and/or progression of geriatric neuropsychiatric disorders.

## Introduction

Coronavirus Disease 2019 (COVID-19, named by WHO on Feb 11, 2020) outbreak was officially reported in December 2019 in Wuhan, Hubei Province, China, and rapidly reached a pandemic status (1–3). The COVID-19 is caused by a novel positive-sense single-stranded RNA virus known as severe acute respiratory syndrome coronavirus 2 (SARS-CoV-2) (2, 4). Similar to other coronavirus like the SARS-CoV-1, the novel SARS-CoV-2 primarily targets the human respiratory system and may cause severe pneumonia and ultimately death. The mortality rate ranges from 2 to 4% of the cases, being particurlarly high among those beyond the sixth decade of life with cardiovascular (CVD) and metabolic diseases like diabetes (5–7). Less severe clinical manifestations include fever, fatigue, chills, dry cough, rhinorrhoea, sneezing, and sore throat (8, 9).

Apart from the respiratory and systemic symptoms, there is growing evidence that the 2019-nCoV may also affect the central nervous system (CNS). Approximately, 36.4% (78/214) of patients diagnosed with COVID-19 experienced neurological symptoms like dizziness, headache, impaired arousal, ataxia, and seizure. It is worthing noticing that these symptoms were mainly related to other severe symptoms of the disease (10). Anosmia and dysgeusia have also been reported, and may proceed the typical respiratory symptoms (11). The first case of viral meningoencephalitis caused by the 2019-nCoV was reported in a 24-years-old man admitted to a hospital with seizures accompanied by impaired arousal, with the virus genome being identified in the cerebrospinal fluid (CSF) (12). Further evidence provided by systematic reviews and meta-analysis has supported the occurrence of neurological manifestations in patients with COVID-19 (13, 14).

Like the SARS-CoV-1, the SARS-CoV-2 seems to exploit the angiotensin-converting enzyme 2 (ACE2) receptor to entry the host cells (15). The evidence mainly from pre-clinical studies suggesting that ACE inhibitors (ACEIs) and angiotensin receptor blockers (ARBs), drugs often prescribed for CVD, kidney, and metabolic diseases, might up-regulate circulating and tissue expression of ACE2 (16–20), raised the question whether those therapies increase SARS-CoV-2 infectivity and COVID-19 severity. Accordingly, some researches proposed the discontinuation of ACEIs and ARBs, both prophylactically and in the context of suspected Covid-19 (6, 21–23). In a period when all information, especially with alarmist content, spreads fast in social media, this suggestion increased the anxiety among people using those medications. However, expert opinions have discouraged treatment discontinuation due to limited evidence on the potential effects of this strategy in COVID-19 outcomes, and that withdrawal may contribute to clinical decompensation of high-risk patients (24).

In this rapidly evolving scenario, herein, we aim to discuss the use of ACEIs and ARBs in high-risk patients for COVID-19. We propose that beyond the risk of clinical complications with the discontinuation of ACEIs and ARBs, these drugs might exert a potentially protective role against the emergence and/or progression of geriatric neuropsychiatric disorders. To support our proposal, we first address the dilemma of discontinuation of RAS blockers during the COVID-19 pandemic. Second, we review the role of renin-angiotensin system (RAS) components in neurodegenerative and neuropsychiatry disorders. Finally, we discuss the potential protective role of ACEIs and ARBs on the development and/or progression of geriatric neuropsychiatric disorders in high-risk patients for COVID-19.

## ACEIs and ARBs Use During COVID-19: Foe or Friend?

Coexisting conditions such as older age, CVD and diabetes seem to be key prognostic determinants in response to the infection with 2019-nCoV. Severe symptoms of COVID-19 and high mortality have been associated with these conditions (6, 7, 25, 26).

ACEIs and ARBs are frequently prescribed for older adults with CVD, kidney and metabolic diseases. Among multiple biological effects, ACEIs, and ARBs seem to increase the expression of ACE2 (17, 18, 20). The increase in ACE2 expression in response to ACEIs and ARBs treatments has been shown mostly in pre-clinical studies (17–20). There are only few human studies specifically addressing this issue, with conflicting results. The ARB olmesartan increased urinary levels of ACE2 in hypertensive patients (20) and patients with diabetic nephropathy (27). Conversely, no effect in ACE2 urinary levels was found with the ACEI enalapril or other ARBs (losartan, candesartan, valsartan, and telmisartan) (20). Although ACE2 shows a 40% structural homology with ACE, they present a different conformational structure of the catalytic site, which may explain why ACEI in clinical use do not directly affect ACE2 activity or expression (28, 29). The failure of most ARBs in changing ACE2 urinary levels revealed that such effects might not be uniform across RAS blockers even considering the same drug class (20). Moreover, no changes in ACE2 activity was found in the plasma of patients with heart failure, atrial fibrillation, aortic stenosis, and coronary artery disease under ACEIs or ARBs therapy compared with untreated patients (30–33). Importantly, there is no available experimental or clinical evidence regarding the effects of ACEIs or ARBs on the expression of ACE2 in the lung, the primary tissue target by SARS-CoV-2 infection (34).

The recent discovery that the 2019-nCoV uses the renin–angiotensin system (RAS) component ACE2 as a receptor to invade host epithelial cells and cause organs damage, prompted the debate regarding ACEIs and ARBs use during COVID-19 pandemic. Based on the debatable claim that ACEIs and ARBs would increase ACE2 expression in humans, some authors proposed the discontinuation of ACEIs and ARBs for CVD, kidney, and metabolic diseases (6, 21–23). However, in the absence of clinical evidence, professional societies have advocated their continued use (24). Supporting this position, a clinical study conducted at the Central Hospital of Wuhan, China, with 362 hypertension patients hospitalized with COVID-19, demonstrated that ACEIs and ARBs therapies were not associated with increase in COVID-19 severity or mortality. The comparison among patients under ACEI/ARBs combined or monotherapy (115, 31.8%), patients taking other hypertensive drugs, especially calcium-channel blockers (168, 46.4%), or not receiving any drug treatment (65, 18%) showed no significant differences in laboratory results, including blood counts, inflammatory markers, renal, and liver function tests, and cardiac biomarkers, or clinical outcomes (35). Later other studies conducted in selected health care systems in North America (36, 37) and Europe (38, 39) supported the concept that ACEIs and ARBs are not associated with worst clinical outcomes. Taken together, these studies provide convincing evidence against the discontinuation of ACEIs and ARBs use in patients with or at risk for COVID-19.

It is also important to consider the multiple roles of ACE2 as a component of RAS, a cascade of vasoactive peptides that regulates key physiological functions, including blood pressure and hydroelectrolyte balance (40, 41). Apart from acting as a circulating hormonal system, RAS components are locally expressed in several organs and tissues, including kidney, brain, and lung, exerting physiological actions through tissue-specific mechanisms (42, 43). In the RAS pathway, angiotensinogen mainly produced by the liver is cleaved by renin, synthesized by the kidneys, in Ang I (pro-angiotensin). The angiotensin converting enzyme (ACE) cleaves the deca-peptide Ang I to the 8-amino acid peptide Ang II, which exerts its effects mainly through the Ang II type 1 (AT1) receptor. Ang II is also a substrate for ACE2, a cell membrane protein with a 17-amino acids N-terminal signal peptide and a C-terminal membrane anchor that acts as monocarboxypeptidase with a catalytically active ectodomain located at the extracellular side of the cell (44, 45). Importantly, the C-terminal domain of ACE2 shares significant homology with Collectrin, a type I membrane protein highly expressed on renal proximal tubules. Collectrin is involved in the process of vesicle transport and membrane fusion, properties that ACE2 also owns, which may facilitate the use of ACE2 as a receptor for SARS-CoV-2 gain entry in the host cells resulting in COVID-19 (46). As a RAS component, ACE2 directly converts Ang II in the seven-amino-acid heptapeptide Ang-(1-7), which activates G protein-coupled MAS receptor. This type 1 transmembrane glycoprotein also cleaves the C-terminal amino acid of Ang I to the non-peptide Ang-(1-9), which in turn is converted to Ang-(1-7) by ACE and Neprilysin, an enzyme also known as neutral endopeptidase. The catalytic efficiency of ACE2 is 400 times higher on Ang II than on Ang I, favoring the direct production of Ang-(1-7) (44, 45).

The RAS is composed traditionally categorized into two arms: the classical one, including ACE, Angiotensin (Ang) II, Ang type 1 (AT1) receptor (ACE/AngII/AT1), and the “alternative” one, comprising ACE2, Ang-(1-7), Mas receptor (ACE2/Ang1-7/Mas). The classical arm mediates pro-inflammatory, pro-thrombotic, and pro-fibrotic processes, mainly through the activation of AT1 receptors (47). On the other hand, the alternative arm seems to play protective roles by frequently opposing Ang II actions through Mas receptors activation (41, 48, 49). For instance, pre-clinical and clinical evidence revealed that up-regulation of ACE2 expression protects acute lung injury at least in part by decreasing AT1 receptors activation (50). Accordingly, therapeutic strategies have been designed to inhibit ACE/Ang II/AT1 axis and to stimulate ACE2/Ang-(1-7)/Mas receptor activities (41, 51).

SARS-CoV-2 binds to ACE2 in order to gain initial entry in host lung epithelial cells. Theoretically, this process promotes down-regulation of ACE2 expression on epithelial cell surface, which in turn contributes to up-regulation of Ang II inflammatory signaling, enhancing the acute lung injury (52, 53). These findings were observed in a murine model of SARS-CoV-1 induced by administration of Spike (S318-510)-Fc. In this model, acute severe lung injury was associated with decreased tissue expression of ACE2 and enhanced levels of Ang II. Importantly, the ARBs losartan (15 mg/kg) rescued mice from SARS-CoV-1 Spike–mediated lung failure, potentially by restoring ACE2 levels in the lung and favoring the conversion of Ang II in Ang-(1-7) (54). Supporting these findings, administration of the ACE2 agonist diminazene aceturate to mice submitted to hyperoxic lung injury increased lung ACE2 expression/activity and decreased Ang II/Ang-(1-7) ratio, which in turn reduced inflammation and severity of lung failure (55). While no study has replicated these results in experimental models of SARS-CoV-2, elevated plasma levels of Ang II were positively correlated with viral load and lung injury scores in patients diagnosed with COVID-19 (53). Together these results suggest that an imbalance between ACE/AngII/AT1 and ACE2/Ang1-7/Mas axes toward the activation of the former might play a pathophysiological role in COVID-19.

It is worth mentioning that ACE2 levels decline with age, which may predispose to a pro-inflammatory profile as the result of RAS classical arm activation (56, 57). A pro-inflammatory profile also underlies hypertension and diabetes pathophysiology, conditions highly prevalent with aging (57). A possible decrease in ACE2 induced by the SARS-CoV-2 infection in older people, especially those with CVD and diabetes, may exacerbate the pro-inflammatory background, leading to greater COVID-19 severity and mortality (58). Therefore, beyond the risk of clinical decompensation, discontinuation of ACEIs and ARBs is potentially harmful because the subsequent enhanced ACE/Ang II/AT1 receptor activity can worsen inflammatory lung injury and other organs damage. In fact, experimental suppression of ACE2 through genetic deletion or inhibitors was associated with myocardial damage and severe acute lung injury (59–61). Conversely, strategies focused in increasing ACE2 levels or activity such as administration of recombinant human ACE2 (rhACE2), have shown protective effects in CVD and pulmonary diseases (59, 60, 62) and have been suggested as a potential biological therapy against SARS-CoV-2 infection (63, 64).

## ACE2-Angiotensin (1-7)-MAS Receptors Axis Role in Geriatric-Related Neuropsychiatric Disorders

Over the past decades, accumulating evidence has pointed out the role for RAS components in neuropsychiatric disorders [for review see (49, 51, 65, 66)]. Our research group has extensively investigated the profile of RAS molecules in the blood and/or cerebrospinal fluid (CSF) of patients with different neurodegenerative and psychiatric conditions, including Parkinson's disease (67), Alzheimer's disease (AD) (68), and schizophrenia (69). For example, patients with Parkinson's disease presented decreased circulating levels of Ang II and Ang-(1-7) along with increased severity of depressive symptoms (67). Lower CSF levels of ACE were found in patients with AD compared with healthy controls. A significant positive correlation between ACE and Aβ42 levels among patients was also observed, reinforcing the hypothesis that ACE is associated with amyloid-β pathology in AD (68).

The protective effects exerted by ACEIs and ARBs treatments in pre-clinical and clinical settings have supported the involvement of RAS in neuropsychiatric conditions as well (51, 70, 71). In animal models of Parkinson's disease induced by MPTP or 6-hydroxydopamine, administration of AT1 receptor antagonists like losartan and ACEIs such as perindopril prevented motor dysfunction and resulted in increased dopamine striatal levels and neuronal survival (72–76). In a mouse model of AD induced by intracerebroventricular injection of amyloid-β, administration of the ARB telmisartan improved cognitive decline, increased cerebral blood flow, and attenuated brain inflammation and oxidative stress (77). Similar findings were reported following intranasal administration of the ARB losartan in the transgenic APP/PS1 model of AD (78). Epidemiological studies also revealed that ACEIs and ARBs significantly reduced the risk of AD and aging-associated cognitive decline (79). A 6-months treatment with the ARB telmisartan in hypertensive patients with AD resulted in more positive effects in cognition and cerebral blood flow than other anti-hypertensive drugs such as amlodipine (80).

Based on the role of RAS components in regulating hemodynamic functions, a wide range of studies have also supported the involvement of RAS in cerebrovascular diseases, especially stroke (49). Experimental studies with animal models of cerebral ischemia-reperfusion injury demonstrated that central or systemic infusions of Ang II decrease blood flow in the penumbra and increase cerebral inflammation, oxidative stress and edema, which in turn increase stroke-associated mortality (81–84). Administration of the ACEI captopril in rats following hemorrhagic stroke attenuated cerebral herniation and hematoma expansion, prevented new hemorrhage formation, and restored cerebral blood flow regulation (85). Additionally, clinical trials conducted with ARBs including losartan and eprosartan revealed decrease of ~25% in stroke incidence compared with other anti-hypertensive drugs like atenolol and nitrendipine. The effectiveness of ARBs in stroke prevention could not be explained only by blood pressure reduction, indicating that other mechanisms like anti-inflammatory and antioxidant effects may underly their neuroprotection (86, 87).

The expression of RAS components seems to be influenced by ACEIs and ARBs. For instance, patients with hypertension and chronic kidney diseases taking ACEI or ARB presented enhanced circulating levels of Ang-(1-7) (43). As Ang-(1-7) exerts beneficial effects by opposing Ang II actions (41), it is tempting to hypothezise that ACEIs and ARBs neuroprotection may involve in part the activation of the ACE2/ Ang-(1-7) / Mas receptor arm. Supporting this hypothesis, mice overexpressing ACE2 had lower infarct volume and increased cerebral blood flow and neurological function compared to wild type mice. The neuroprotective effects were associated with increased Ang (1-7)/Ang II ratio, angiogenic factors, and attenuated oxidative stress in the brain (88). Moreover, several studies employed pharmacological and/or genetic strategies in order to increase ACE2/Ang-(1-7)/Mas axis activity and revealed protective effects of those RAS components in neuropsychiatric and cerebrovascular conditions [for review see (49, 51, 89)]. For instance, intracerebroventricular infusion of Ang-(1-7) for 4 weeks significantly improved cognitive function and cerebrovascular reactivity in 5XFAD mice, a model of AD (90). Intracerebroventricular infusion of Ang-(1-7) for 2 weeks also prevented cognitive decline and decrease the expression of hippocampal phospho-tau, amyloid-ß oligomer, and both soluble (Aβ 1-42) and insoluble (Aβ 1-40) ß- amyloid peptide in an AD-like rat model resulting from streptozotocin-induced diabetes. Importantly, the beneficial effects of Ang-(1-7) infusion were hampered by the coadministration of A-779, an antagonist of Mas receptors, suggesting that Ang-(1-7) protective activity was mediated by the activation of Mas receptors (91). A more recent study provided evidence that ACE2 activation also exerts protective effects in a transgenic mouse model of AD. Chronic intraperitoneal administration of DIZE (15 mg/kg/day), an established activator of ACE2, restored cognitive decline in Tg25676 mice, which was associated with reduced hippocampal levels of soluble Aβ 1-42 and of pro-inflammatory mediator IL-1ß alongside increased expression of Mas receptor. DIZE also reinstated the balance of hippocampal RAS activity, by increasing the ACE2/ACE activity ratio. Moreover, DIZE-mediated protection was abolished when co-administered with C16, an ACE2 inhibitor, indicating that neuroprotective effects resulted specifically from the enhancement of ACE2 activity (92).

Finally, a post-mortem study revealed that low activity of ACE2 in mid-frontal cortex of patients with AD negatively correlated with Aβ expression and phosphorylated tau pathology. The ratio of Ang II to Ang (1-7) was also reduced in the brain of patients compared with age-matched non-demented controls (93), while low circulating levels of Ang-(1-7) correlated with cognitive decline severity in patients with AD (94). The inverse correlation between concentrations of Ang-(1-7) and tau hyperphosphorylation was also reported in the cerebral cortex and hippocampus of the senescence-accelerated mouse prone 8 (SAMP8) mice, a model of sporadic AD and of the P301S mice, an animal model of tauopathy (95). Taken together these studies corroborate the view that potentiating the systemic or local expression of ACE2 and/or Ang-(1-7) is potentially beneficial under several pathological conditions.

Apart from ACE2, Neprilysin or neutral endopeptidase, a type II membrane protein that belongs to the family of zinc dependent metalloproteases and is expressed in several tissues such as kidney, brain, heart, and lungs, also potentiates the alternative arm of RAS (ACE2/Ang1-7/Mas receptors) by converting Ang-(1-9) in Ang-(1-7) (44). The combination of anti-hypertensive drugs like ARBs with neprilysin inhibitors seems to be protective in hypertension, a risk factor for AD (96). Based on the fact that Neprilysin plays a pivotal role as an amyloid β peptide (Aβ)- degrading enzyme (97), the effect of Neprilysin in AD goes in opposite direction. Therefore, an anti-AD therapeutic strategy should rely on potentiating Neprilysin actions (96). It is worth mentioning that to the best of our knowledge, no strategy combining Neprilysin activation with RAS blockers has been investigated as a potential treatment for AD.

## Discussion: Challenges and Opportunities

ACEIs and ARBs are commonly prescribed for hypertension, myocardial infarction, heart failure, and diabetic nephropathy (98–100). The discovery of the expression of RAS components in the brain stimulated the investigation of the potential effects of ACE inhibition and AT1 receptor antagonism on the physiopathology of neuropsychiatric disorders (71).

A low-grade pro-inflammatory profile has been associated with aging, a process that has been called “inflammaging” (101). This pro-inflammatory profile has been associated with late-life depression and neurocognitive disorders, and increased risk for the development of neurodegenerative diseases (101–103). For instance, patients with mild neurocognitive disorder who progressed to major neurocognitive disorder had significantly higher baseline levels of inflammatory mediators compared to those who retained the diagnosis of mild neurocognitive disorder on follow-up (104).

Recent studies have shown that SARS-CoV-2 can induce a severe systemic inflammatory response, which has been associated with multiple organ failure and, as consequence, a large number of fatalities (105–107). Increased circulating levels of interleukin (IL)-6 were positively correlated with pneumonia severity in patients diagnosed with COVID-19 (107). It is worth mentioning that cytokines like IL-6 are important mediators of the continuous cross-talk between the periphery and the brain (108). Increased levels of systemic cytokines can lead to cognitive and behavioral changes in response to viral infections like influenza (109–112). Mice infected intranasally with live influenza A/PR8/34 (H1N1) exhibited loss of body weight, decreased locomotor activity, and hippocampal-dependent memory impairment. Behavioral and cognitive symptoms were associated with enhanced mRNA expression of inflammatory cytokines (IL-1β, IL-6, IFN-α, and TNF-α) alongside increased microglia reactivity and alterations in neuronal architecture in the hippocampus (111, 112). Patients with influenza-associated acute encephalopathy/encephalitis exhibited neurological symptoms like seizure, altered arousal, and abnormal behaviors, which were associated with increased concentrations of IL-1β, IL-6, and TNF-α in serum and CSF (110). High serum levels of IL-6 in patients with influenza virus-associated encephalopathy were associated with poor clinical prognosis including neurological sequelae and death (109). To date these effects have not been systematically reported and/or studied in the COVID-19 as all efforts have been dedicated to battle the epidemic and minimize its death toll. Therefore, this unchartered area must be explored.

It is highly possible that, after the epidemic is controlled or over, post-COVID-19 neuropsychiatric conditions, notably neurocognitive disorders, will be unveiled. As older adults with CVD and/or diabetes display a more intense pro-inflammatory profile than older adults without these comorbidities (56, 57), and seem to be more vulnerable to the systemic inflammation induced by SARS-CoV-2 (58), they are at higher risk of CNS dysfunction and neurodegeneration as well. Besides these indirect effects, SARS-CoV-2 may also invade the CNS through the olfactory trait, but it remains to be established whether the virus can directly damage neurons and glial cells (113, 114).

**Figure 1:**
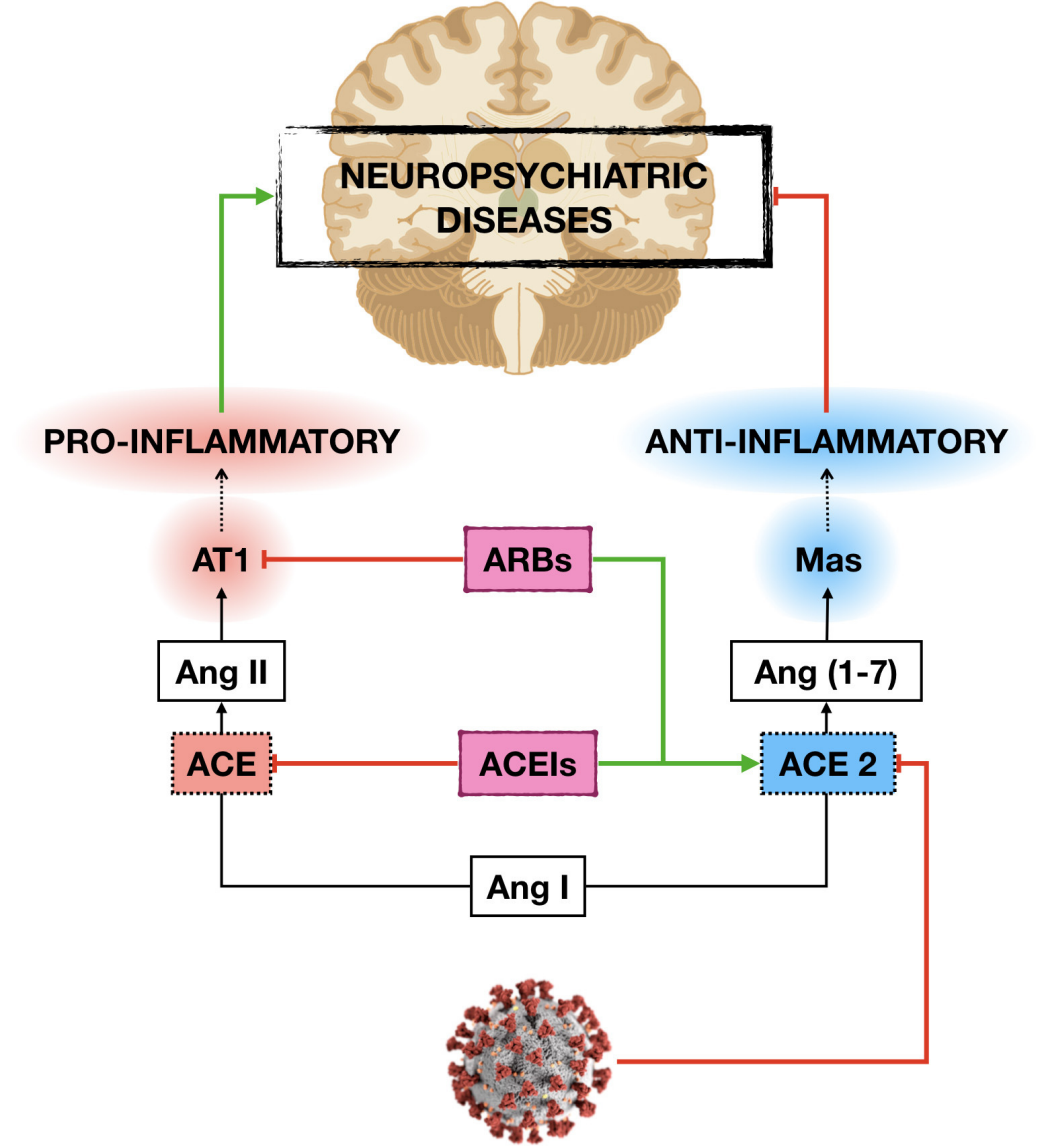
Potential mechanisms by which ACE inhibitors (ACEIs) and angiotensin receptor blockers (ARBs) may protect the development and/or progression of neuropsychiatric diseases in older adults with cardiovascular (CVD) and metabolic diseases during COVID-19 pandemic. By increasing the expression of angiotensin-converting enzyme 2 (ACE2), ACEIs, and ARBs decrease angiotensin II (Ang II) inflammatory signaling and vascular damage induced by the novel severe acute respiratory syndrome coronavirus 2 (SARS-CoV-2) infection, which in turn may protect the central nervous system damage.

The neuroprotective effects of ACEIs and ARBs seem to rely on the anti-inflammatory response exerted by the activation of ACE2/Ang-(1-7) /Mas axis and the decrease in Ang-II inflammatory signaling (51, 89). Therefore, the anti-inflammatory effects induced by ACEIs and ARBs may constitute a protective mechanism not only for the lung but also for other organs, including the brain, especially at high-risk subjects as older adults with comorbities (Figure 1).

Respiratory-related infections like influenza seem to be an independent risk factor for stroke (115–117). Patients with severe symptoms of COVID-19 presented elevated levels of D-dimer and significant platelet reduction, which also pedispose these patients to acute cerebrovascular events (118). As ACE2 is a cardio-cerebral vascular protection molecule (41, 49), the dysregulation of ACE2 induced by SARS-CoV-2 infection may lead to abnormally elevated blood pressure and increase the risk of cerebral hemorrhage. Moreover, by binding to ACE2 receptors expressed on the capillary endothelium, the SARS-CoV-2 may disrupt the blood-brain barrier and get access into the CNS (113). ACEIs and ARBs may prevent or attenuate the deleterious vascular events induced by the SARS-CoV-2, also minimizing its potential damage to CNS in older adults.

Another aspect that must be considered during the COVID-19 pandemic is the development of post-traumatic stress disorder (PTSD). Besides emotional aspects including fear to be ill or die as well as the impact of isolation may significantly account for the development of PTSD, neurobiological factors must be taken into account. PTSD has been associated with CVD and changes in the RAS (119, 120). For instance, a cross-sectional study revealed that patients diagnosed with PTSD undergoing ACEIs or ARBs treatment for hypertension presented less PTSD symptoms including hyperarousal symptoms, avoidance, and intrusive thoughts when compared with PTSD patients not on ACEIs or ARBs treatments. Of note, other anti-hypertensive drugs, including beta-blockers, calcium channel blockers, and diuretics, were not significantly associated with reduced PTSD symptoms (119). This is particularly relevant for high-risk patients taking ACEIs and/or ARBs, further supporting the potential harmful effects of discontinuation of these drugs during the treatment of COVID-19. It remains to be answered whether there is any benefit of prescribing ACEIs or ARBs for older adults not taking these medications in order to minimize the complications related to the COVID-19, including the neuropsychiatric ones.

## Concluding Remarks

The discovery that SARS-CoV-2, the virus responsible for the COVID-19, enters the host cells by binding ACE2 receptors, generated a debate regarding the discontinuation or not of ACEIs and ARBs in patients with CVD and diabetes. These comorbities are prevalent among older adults, also being associated with COVID-19 severity and mortality. At first, some authors proposed the discontinuation of ACEIs and ARBs based on the evidence that those drugs can enhance ACE2 levels, supposedly facilitating virus infection. However, this generated a significant backlash with expert opinions recommending against the discontinuation due to the lack of empirical evidence to support the proposal and the potential risk of cardiovascular complications. Moreover, from a theoretical perspective, ACEIs and ARBs may stimulate the anti-inflammatory properties of ACE2/Ang-(1-7)/Mas axis and, therefore, improve COVID-19 associated severity and mortality.

Besides potentially inducing severe systemic inflammatory response, SARS-CoV-2 also seems to present neurotropism, although the exact extension and mechanisms by which the virus affect the CNS is unclear. In this scenario of enhanced systemic inflammation and potential neuroinflammation, older adults, especially those with CVD and diabetes, are more likely to develop cognitive and behavioral changes alongside neurodegenerative diseases in response to SARS-CoV-2 infection. Given the vascular and anti-inflammatory properties of ACE2 and Ang (1-7), beneficial not harmful effects are expected from ACEIs and ARBs, so these medications should not be withdrawn in older adults.

## Author Contributions

AM drafted the initial version of the manuscript that was revised and modified by AT. All authors have read and approved the final version of the manuscript.

## Conflict of Interest

AT is a CNPq fellowship recipient while AM is a 2019 “For Women in Science” Grant Awardee from the L'Oreal Brazil-UNESCO- Brazilian Academy of Science (ABC).

## References

[1] Li Q Guan X Wu P Wang X Zhou L Tong Y. Early transmission dynamics in Wuhan, China, of novel coronavirus-infected pneumonia. N Engl J Med. (2020) 382:1199–207. 10.1056/NEJMoa2001316 31995857 PMC7121484

[2] Lu H Stratton CW Tang YW. Outbreak of pneumonia of unknown etiology in Wuhan China: the mystery and the miracle. J Med Virol. (2020) 92:401–2. 10.1002/jmv.25678 31950516 PMC7166628

[3] Thompson R. Pandemic potential of 2019-nCoV. Lancet Infect Dis. (2020) 20:280. 10.1016/S1473-309930068-2 32043978 PMC7128127

[4] Rothan HA Byrareddy SN. The epidemiology and pathogenesis of coronavirus disease (COVID-19) outbreak. J Autoimmun. (2020) 26:102433. 10.1016/j.jaut.2020.102433 32113704 PMC7127067

[5] Bassetti M Vena A Giacobbe DR. The Novel Chinese Coronavirus (2019-nCoV) infections: challenges for fighting the storm. Eur J Clin Invest. (2020) 50:e13209. 10.1111/eci.13209 32003000 PMC7163647

[6] Fang L Karakiulakis G Roth M. Are patients with hypertension and diabetes mellitus at increased risk for COVID-19 infection? Lancet Respir Med. (2020) 8:e21. 10.1016/S2213-260030116-8 32171062 PMC7118626

[7] Ren LL Wang YM Wu ZQ Xiang ZC Guo L Xu T. Identification of a novel coronavirus causing severe pneumonia in human: a descriptive study. Chinese Med J. (2020) 133:1015–24. 10.1097/CM9.0000000000000722 32004165 PMC7147275

[8] Guan W Ni Z Hu Y Liang WH Ou CQ He JX Clinical characteristics of coronavirus disease 2019 in China. N Engl J Med. (2020) 382:1708–20. 10.1056/NEJMoa2002032 32109013 PMC7092819

[9] Huang C Wang Y Li X Ren L Zhao J Hu Y. Clinical features of patients infected with 2019 novel coronavirus in Wuhan, China. Lancet. (2020) 395:497–506. 10.1016/S0140-673630183-5 31986264 PMC7159299

[10] Mao L Wang MD Chen SH He Q Chang J Hong C. Neurological manifestations of hospitalized patients with COVID-19 in Wuhan, China: a retrospective case series study. MedRxiv. 10.2139/ssrn.354484032275288

[11] Giacomelli A Pezzati L Conti F Bernacchia D Siano M Oreni L Self-reported olfactory and taste disorders in SARS-CoV-2 patients: a cross-sectional study. Clin Infect Dis. (2020) 71:889–90. 10.1093/cid/ciaa330 32215618 PMC7184514

[12] Moriguchi T Harii N Goto J Harada D Sugawara H Takamino J. A first Case of Meningitis/Encephalitis associated with SARS-Coronavirus-2. Int J Infect Dis. (2020) 94:55–8. 10.1016/j.ijid.2020.03.062 32251791 PMC7195378

[13] Tsai ST Lu MK San S Tsai CH. The neurologic manifestations of coronavirus disease 2019 pandemic: a systemic review. Front Neurol. (2020) 11:498. 10.3389/fneur.2020.00498 32574246 PMC7248254

[14] Wang L Shen Y Li M Chuang H Ye Y Zhao H. Clinical manifestations and evidence of neurological involvement in 2019 novel coronavirus SARS-CoV-2: a systematic review and meta-analysis. J Neurol. (2020). 10.1007/s00415-020-09974-2. [Epub ahead of print]. 32529575 PMC7288253

[15] Hoffmann M Kleine-Weber H Schroeder S Krüger N Herrler T Erichsen S. SARS- CoV-2 cell entry depends on ACE2 and TMPRSS2 and is blocked by a clinically proven protease inhibitor. Cell. (2020) 181:271–80.e8. 10.1016/j.cell.2020.02.052 32142651 PMC7102627

[16] Ferrario CM Jessup J Chappell MC Averill DB Brosnihan KB Tallant EA. Effect of angiotensin-converting enzyme inhibition and angiotensin II receptor blockers on cardiac angiotensin-converting enzyme 2. Circulation. (2005) 111:2605–10. 10.1161/CIRCULATIONAHA.104.510461 15897343

[17] Ocaranza MP Godoy I Jalil JE Varas M Collantes P Pinto M. Enalapril attenuates downregulation of angiotensin-converting enzyme 2 in the late phase of ventricular dysfunction in myocardial infarcted rat. Hypertension. (2006) 48:572–8. 10.1161/01.HYP.0000237862.94083.45 16908757

[18] Hamming I van Goor H Turner AJ Rushworth CA Michaud AA Corvol P. Differential regulation of renal angiotensin-converting enzyme (ACE) and ACE2 during ACE inhibition and dietary sodium restriction in healthy rats. Exp Physiol. (2008) 93:631–8. 10.1113/expphysiol.2007.041855 18192334

[19] Soler MJ Barrios C Oliva R Batlle D. Pharmacologic modulation of ACE2 expression. Curr Hypertens Rep. (2008) 10:410–4. 10.1007/s11906-008-0076-0 18775121 PMC7089438

[20] Furuhashi M Moniwa N Mita T Fuseya T Ishimura S Ohno K. Urinary angiotensin- converting enzyme 2 in hypertensive patients may be increased by olmesartan, an angiotensin II receptor blocker. Am J Hypertens. (2015) 28:15–21. 10.1093/ajh/hpu086 24842388

[21] Diaz JH. Hypothesis: angiotensin-converting enzyme inhibi- tors and angiotensin receptor blockers may increase the risk of severe COVID-19. J Travel Med. (2020) 27:taaa041. 10.1093/jtm/taaa041 32186711 PMC7184445

[22] Esler M Esler D. Can angiotensin receptor-blocking drugs perhaps be harmful in the COVID-19 pandemic? J Hypertens. (2020) 38:781–2. 10.1097/HJH.0000000000002450 32195824

[23] Sommerstein R Gräni C Preventing a COVID-19 pandemic: ACE inhibitors as a potential risk factor for fatal COVID-19. BMJ. (2020) 368:m810 10.1136/bmj.m810 32111649

[24] Saavedra JM. Angiotensin receptor blockers 2019-nCoV. Pharmacol Res. (2020) 156:104832. 10.1016/j.phrs.2020.104832 32304747 PMC7158830

[25] Yang J Zheng Y Gou X Ke Pu Zhaofeng C Qinghong G. Prevalence of comorbidities and its effects in patients infected with SARS-CoV-2: a systematic review and meta-analysis. Int J Infect Dis. (2020) 94:91–5. 10.1016/j.ijid.2020.03.017 32173574 PMC7194638

[26] Zhou F Yu T Du R Fan G Liu Y Liu Z Clinical course and risk factors for mortality of adult inpatients with COVID-19 in Wuhan, China: a retrospective cohort study. Lancet. (2020) 395:1054–62. 10.1016/S0140-673630566-3 32171076 PMC7270627

[27] Abe M Oikawa O Okada K Soma M. Urinary angiotensin-converting enzyme 2 increases in diabetic nephropathy by angiotensin II type 1 receptor blocker olmesartan. J Renin Angiotensin Aldosterone Syst. (2015) 16:159–64. 10.1177/1470320314551443 25287898

[28] Tipnis SR Hooper NM Hyde R Karran E Christie G Turner AJ. A human homolog of angiotensin-converting enzyme. Cloning and functional expression as a captopril-insensitive carboxypeptidase. J Biol Chem. (2000) 275:33238–43. 10.1074/jbc.M002615200 10924499

[29] Rice GI Thomas DA Grant PJ Turner AJ Hooper NM. Evaluation of angiotensin-converting enzyme (ACE), its homologue ACE2 and neprilysin in angiotensin peptide metabolism. Biochem J. (2004) 383:45–51. 10.1042/BJ20040634 15283675 PMC1134042

[30] Epelman S Shrestha K Troughton RW Francis GS Sen S Klein AL. Soluble angio- tensin-converting enzyme 2 in human heart failure: relation with myocardial function and clinical outcomes. J Card Fail. (2009) 15:565–71. 10.1016/j.cardfail.2009.01.014 19700132 PMC3179261

[31] Walters TE Kalman JM Patel SK Mearns M Velkoska E Burrell LM. Angiotensin converting enzyme 2 activity and human atrial fibrillation: increased plasma angiotensin converting enzyme 2 activity is associated with atrial fibrillation and more advanced left atrial structural remodelling. Europace. (2017) 19:1280–7. 10.1093/europace/euw246 27738071

[32] Ramchand J Patel SK Kearney LG Matalanis G Farouque O Srivastava PM. Plasma ACE2 activity predicts mortality in aortic stenosis and is associated with severe myocardial fibrosis. JACC Cardiovasc Imaging. (2020) 13:655–64. 10.1016/j.jcmg.2019.09.005 31607667

[33] Ramchand J Patel SK Srivastava PM Farouque O Burrell LM Elevated plasma angiotensin converting enzyme 2 activity is an independent predictor of major adverse cardiac events in pa- tients with obstructive coronary artery disease. PLoS ONE. (2018) 13:e0198144 10.1371/journal.pone.0198144 29897923 PMC5999069

[34] Vaduganathan M Vardeny O Michel T McMurray JJV Pfeffer MA Solomon SD. Renin–angiotensin–aldosteronesysteminhibitorsin patients with COVID-19. N Engl J Med. (2020) 382:1653–9. 10.1056/NEJMsr2005760 32227760 PMC7121452

[35] Li J Wang X Chen J Zhang H Deng A Association of renin-angiotensin system inhibitors with severity or risk of death in patients with hypertension hospitalized for coronavirus disease 2019 (COVID-19) infection in Wuhan, China. JAMA Cardiol. (2020) 5:1–6. 10.1001/jamacardio.2020.1624 PMC7180726 32324209

[36] Mehta N Kalra A Nowacki AS Anjewierden S Han Z Bhat P. Association of use of angiotensin-converting enzyme inhibitors and angiotensin ii receptor blockers with testing positive for coronavirus disease 2019 (COVID-19). JAMA Cardiol. (2020) e201855. 10.1001/jamacardio.2020.1855 32936273 PMC7201375

[37] Reynolds HR Adhikari S Pulgarin C Troxel AB Iturrate E Johnson SB. Renin–angiotensin–aldosterone system inhibitors and risk of Covid-19. N Engl J Med. (2020) 382:2441–8. 10.1056/NEJMoa2008975 32356628 PMC7206932

[38] Fosbøl EL Butt JH Østergaard L Andersson C Selmer C Kragholm K Association of angiotensin-converting enzyme inhibitor or angiotensin receptor blocker use with COVID-19 diagnosis and mortality. JAMA. (2020) 324:168–77. 10.1001/jama.2020.11301 PMC7305566 32558877

[39] Mancia G Rea F Ludergnani M Apolone G Corrao G. Renin-angiotensin-aldosterone system blockers and the risk of COVID-19. N Engl J Med. (2020) 382:2431–40. 10.1056/NEJMoa2006923 32356627 PMC7206933

[40] Paul M Poyan Mehr A Kreutz R Physiology of local renin-angiotensin systems. Physiol Rev. (2006) 86:747–803. 10.1152/physrev.00036.2005 16816138

[41] Prestes TR Rocha NP Miranda AS Teixeira AL Simoes-E-Silva AC. The Anti-inflammatory potential of ACE2/angiotensin-(1-7)/mas receptor axis: evidence from basic and clinical research. Curr Drug Targets. (2017) 18:1301–13. 10.2174/1389450117666160727142401 27469342

[42] Simoes e Silva AC Flynn JT. The renin-angiotensin-aldosterone system in 2011: role in hypertension and chronic kidney disease. Pediatr Nephrol. (2012) 27:1835–45. 10.1007/s00467-011-2002-y 21947887

[43] Simoes e Silva AC Teixeira MM. ACE inhibition, ACE2 and angiotensin-(1-7) axis in kidney and cardiac inflammation and fibrosis. Pharmacol Res. (2016) 107:154–62. 10.1016/j.phrs.2016.03.018 26995300

[44] Vickers C Hales P Kaushik V Dick L Gavin J Tang J. Hydrolysis of biological peptides by human angiotensin-converting enzyme-related carboxypeptidase. J Biol Chem. (2002) 277:14838–43. 10.1074/jbc.M200581200 11815627

[45] Wang K Gheblawi M Oudit GY. Angiotensin converting enzyme 2: a double- edged sword. Circulation. (2020). 10.1161/CIRCULATIONAHA.120.047049. [Epub ahead of print]. 32213097

[46] Yan R Zhang Y Li Y Xia L Guo Y Zhou Q. Structural basis for the recognition of SARS-CoV-2 by full-length human ACE2. Science. (2020) 367:1444–8. 10.1126/science.abb2762 32132184 PMC7164635

[47] Kamo T Akazawa H Komuro I. Pleiotropic effects of angiotensin II receptor signaling in cardiovascular homeostasis and aging. Int Heart J. (2015) 56:249–54. 10.1536/ihj.14-429 25912907

[48] Simoes e Silva AC Silveira KD Ferreira AJ Teixeira MM ACE2, angiotensin-(1-7) and Mas receptor axis in inflammation and fibrosis. Br J Pharmacol. (2013) 169:477–92. 10.1111/bph.12159 23488800 PMC3682698

[49] Kangussu LM Marzano LAS Souza CF Dantas CC Miranda AS Simões E Silva AC. The renin-angiotensin system and the cerebrovascular diseases: experimental and clinical evidence. Protein Pept Lett. (2019) 27:463–75. 10.2174/0929866527666191218091823 31849284

[50] Jia H. Pulmonary Angiotensin-Converting Enzyme 2 (ACE2) and inflammatory lung disease. Shock. (2016) 46:239–48. 10.1097/SHK.0000000000000633 27082314

[51] Rocha NP Simoes E Silva AC Prestes TRR Feracin V Machado CA Ferreira RN. RAS in the central nervous system: potential role in neuropsychiatric disorders. Curr Med Chem. (2018) 25:3333–52. 10.2174/0929867325666180226102358 29484978

[52] Gurwitz D. Angiotensin receptor blockers as tentative SARS CoV-2 therapeutics. Drug Dev Res. (2020). 10.1002/ddr.21656. [Epub ahead of print]. 32129518 PMC7228359

[53] Liu Y Yang Y Zhang C Huang F Wang F Yuan J. Clinical and biochemical indexes from 2019-nCoV infected patients linked to viral loads and lung injury. Sci China Life Sci. (2020) 63:364–74. 10.1007/s11427-020-1643-8 32048163 PMC7088566

[54] Kuba K Imai Y Rao S Gao H Guo F Guan B. A crucial role of angiotensin converting enzyme 2 (ACE2) in SARS coronavirus-induced lung injury. Nat Med. (2005) 11:875–9. 10.1038/nm1267 16007097 PMC7095783

[55] Fang Y Gao F Liu Z. Angiotensin-converting enzyme 2 attenuates inflammatory response and oxidative stress in hyperoxic lung injury by regulating NF-κB and Nrf2 pathways. QJM. (2019) 112:914–24. 10.1093/qjmed/hcz206 31393582

[56] Xie X Chen J Wang X Zhang F Liu Y. Age-and gender-related difference of ACE2 expression in rat lung. Life Sci. (2006) 78:2166–71. 10.1016/j.lfs.2006.09.028 16303146 PMC7094566

[57] Lakatta EG. The reality of getting old. Nat Rev Cardiol. (2018) 15:499–500. 10.1038/s41569-018-0068-y 30065260

[58] AlGhatrif M Cingolani O Lakatta EG. The dilemma of coronavirus disease 2019, aging, and cardiovascular disease: insights from cardiovascular aging science. JAMA Cardiol. (2020). 10.1001/jamacardio.2020.1329. [Epub ahead of print]. 32242886 PMC10089230

[59] Imai Y Kuba K Rao S Huan Y Guo F Guan B. Angiotensin-converting enzyme 2 protects from severe acute lung failure. Nature. (2005) 436:112–6. 10.1038/nature03712 16001071 PMC7094998

[60] Zhong J Basu R Guo D Chow FL Byrns S Schuster M. Angiotensin-converting enzyme 2 suppresses pathological hypertrophy, myocardial fibrosis, and cardiac dysfunction. Circulation. (2010) 122:717–28. 10.1161/CIRCULATIONAHA.110.955369 20679547

[61] Patel VB Zhong JC Grant MB Oudit GY. Role of the ACE2/angiotensin 1-7 axis of the renin-angiotensin system in heart failure. Circ Res. (2016) 118:1313–26. 10.1161/CIRCRESAHA.116.307708 27081112 PMC4939482

[62] Khan A Benthin C Zeno B Albertson TE Boyd J Christie JD. A pilot clinical trial of recombinant human angiotensin-converting enzyme 2 in acute respiratory distress syndrome. Crit Care. (2017) 21:234. 10.1186/s13054-017-1823-x 28877748 PMC5588692

[63] Batlle D Wysocki J Satchell K. Soluble angiotensin-converting enzyme 2: a potential approach for coronavirus infection therapy? Clin Sci. (2020) 134:543–5. 10.1042/CS20200163 32167153

[64] Roshanravan N Ghaffari S Hedayati M. Angiotensin converting enzyme-2 as therapeutic target in COVID-19. Diabetes Metab Syndr. (2020) 14:637–9. 10.1016/j.dsx.2020.05.022 32428864 PMC7214324

[65] Mohite S Sanches M Teixeira AL. Exploring the evidence implicating the renin- angiotensin system (RAS) in the physiopathology of mood disorders. Protein Pept Lett. (2019) 27:449–55. 10.2174/0929866527666191223144000 31868144

[66] Sassi KLM Martins LB Miranda AS Teixeira AL. Renin-angiotensin-aldosterone system and migraine: a systematic review of human studies. Protein Pept Lett. (2020) 27:512–9. 10.2174/0929866527666200129160136 31995000

[67] Rocha NP Scalzo PL Barbosa IG de Campos-Carli SM Tavares LD de Souza MS. Peripheral levels of angiotensins are associated with depressive symptoms in Parkinson's disease. J Neurol Sci. (2016) 368:235–9. 10.1016/j.jns.2016.07.031 27538640

[68] Rocha NP Toledo A Corgosinho LTS de Souza LC Guimarães HC Resende EPF. Cerebrospinal fluid levels of angiotensin-converting enzyme are associated with amyloid-β42 burden in Alzheimer's disease. J Alzheimers Dis. (2018) 64:1085–90. 10.3233/JAD-180282 30040721

[69] Mohite S de Campos-Carli SM Rocha NP Sharma S Miranda AS Barbosa IG. Lower circulating levels of angiotensin-converting enzyme (ACE) in patients with schizophrenia. Schizophr Res. (2018) 202:50–4. 10.1016/j.schres.2018.06.023 29925475

[70] Villapol S Saavedra JM. Neuroprotective effects of angiotensin receptor blockers. Am J Hypertens. (2015) 28:289–99. 10.1093/ajh/hpu197 25362113

[71] Wincewicz D Braszko JJ. Validation of brain angiotensin system blockade as a novel drug target in pharmacological treatment of neuropsychiatric disorders. Pharmacopsychiatry. (2017) 50:233–47. 10.1055/s-0043-112345 28641333

[72] Kurosaki R Muramatsu Y Kato H Watanabe Y Imai Y Itoyama Y. Effect of angiotensin-converting enzyme inhibitor perindopril on interneurons in MPTP- treated mice. Eur Neuropsychopharmacol. (2005) 15:57–67. 10.1016/j.euroneuro.2004.05.007 15572274

[73] Munoz A Rey P Guerra MJ Mendez-Alvarez E Soto-Otero R Labandeira-Garcia JL. Reduction of dopaminergic degeneration and oxidative stress by inhibition of angiotensin converting enzyme in a MPTP model of parkinsonism. Neuropharmacology. (2006) 51:112–20. 10.1016/j.neuropharm.2006.03.004 16678218

[74] Grammatopoulos TN Jones SM Ahmadi FA Hoover BR Snell LD Skoch J Angiotensin type 1 re- ceptor antagonist losartan, reduces MPTP-induced degeneration of dopaminergic neurons in substantia nigra. Mol Neurodegener. (2007) 2:1 10.1186/1750-1326-2-1 17224059 PMC1783655

[75] Rey P Lopez-Real A Sanchez-Iglesias S Muñoz A Soto-Otero R Labandeira-Garcia JL. Angiotensin type- 1-receptor antagonists reduce 6-hydroxydopamine toxicity for dopaminergic neurons. Neurobiol Aging. (2007) 28:555–67. 10.1016/j.neurobiolaging.2006.02.018 16621167

[76] Munoz A Garrido-Gil P Dominguez-Meijide A Labandeira-Garcia JL. Angiotensin type 1 receptor blockage reduces l-dopa-induced dyskinesia in the 6-OHDA model of Parkinson's disease. Involvement of vascular endothelial growth factor and interleukin-1beta. Exp Neurol. (2014) 261:720–32. 10.1016/j.expneurol.2014.08.019 25160895

[77] Tsukuda K Mogi M Iwanami J Min LJ Sakata A Jing F. Cognitive deficit in amyloid-beta-injected mice was improved by pretreatment with a low dose of telmisartan partly because of peroxisome proliferator-activated receptor-gamma activation. Hypertension. (2009) 54:782–7. 10.1161/HYPERTENSIONAHA.109.136879 19635982

[78] Danielyan L Klein R Hanson LR Buadze M Schwab M Gleiter CH. Protective effects of intranasal losartan in the APP/PS1 transgenic mouse model of Alzheimer disease. Rejuvenation Res. (2010) 13:195–201. 10.1089/rej.2009.0944 20370487

[79] Zhuang S Wang HF Wang X Li J Xing CM. The association of renin-angiotensin system blockade use with the risks of cognitive impairment of aging and Alzheimer's disease: a meta-analysis. J Clin Neurosci. (2016) 33:32–8. 10.1016/j.jocn.2016.02.036 27475317

[80] Kume K Hanyu H Sakurai H Takada Y Onuma T Iwamoto T. Effects of telmisartan on cognition and regional cerebral blood flow in hypertensive patients with Alzheimer's disease. Geriatr Gerontol Int. (2012) 12:207–14. 10.1111/j.1447-0594.2011.00746.x 21929736

[81] Kazama K Anrather J Zhou P Girouard H Frys K Milner TA. Angiotensin II impairs neurovascular coupling in neocortex through NADPH oxidase-derived radicals. Circ Res. (2004) 95:1019–26. 10.1161/01.RES.0000148637.85595.c5 15499027

[82] De Silva TM Broughton BR Drummond GR Sobey CG Miller AA. Gender influences cerebral vascular responses to angiotensin II through Nox2-derived reactive oxygen species. Stroke. (2009) 40:1091–7. 10.1161/STROKEAHA.108.531707 19211495

[83] Jackman KA Miller AA Drummond GR Sobey CG. Importance of NOX1 for angiotensin II-induced cerebrovascular superoxide production and cortical infarct volume following ischemic stroke. Brain Res. (2009) 1286:215–20. 10.1016/j.brainres.2009.06.056 19559686

[84] Mogi M Horiuchi M. Effect of angiotensin II type 2 receptor on stroke, cognitive impairment and neurodegenerative diseases. Geriatr Gerontol Int. (2013) 13:13–8. 10.1111/j.1447-0594.2012.00900.x 22726823

[85] Smeda JS Watson D Stuckless J Negandhi A. Post-stroke losartan and captopril treatments arrest hemorrhagic expansion in SHRsp without lowering blood pressure. Vascul Pharmacol. (2018) 111:26–35. 10.1016/j.vph.2018.08.006 30114508

[86] Dahlöf B Devereux RB Kjeldsen SE Julius S Beevers G de Faire U LIFE Study Group. Cardiovascular morbidity and mortality in the Losartan Intervention for Endpoint reduction in hypertension study (LIFE): a randomised trial against atenolol. Lancet. (2002) 359:995–1003. 10.1016/S0140-673608089-3 11937178

[87] Schrader J Lüders S Kulschewski A Hammersen F Plate K Berger J. MOSES Study Group. Morbidity and mortality after stroke, eprosartan compared with nitrendipine for secondary prevention: principal results of a prospective randomized controlled study (MOSES). Stroke. (2005) 36:1218–26. 10.1161/01.STR.0000166048.35740.a9 15879332

[88] Chen J Zhao Y Chen S Wang J Xiao X Ma X. Neuronal over-expression of ACE2 protects brain from ischemia-induced damage. Neuropharmacology. (2014) 79:550–8. 10.1016/j.neuropharm.2014.01.004 24440367 PMC3992949

[89] Ribeiro VT de Souza LC Simões E Silva AC. Renin-angiotensin system and Alzheimer's disease pathophysiology: from the potential interactions to therapeutic perspectives. Protein Pept Lett. (2019) 27:484–511. 10.2174/0929866527666191230103739 31886744

[90] Uekawa K Hasegawa Y Senju S Nakagata N Ma M Nakagawa T. Intracerebroventricular infusion of angiotensin-(1-7) ameliorates cognitive impairment and memory dysfunction in a mouse model of Alzheimer's disease. J Alzheimers Dis. (2016) 53:127–33. 10.3233/JAD-150642 27128367

[91] Chen JL Zhang DL Sun Y Zhao YX Zhao KX Pu D. Angiotensin-(1-7) administration attenuates Alzheimer's disease-like neuropathology in rats with streptozotocin-induced diabetes via Mas receptor activation. Neuroscience. (2017) 346:267–77. 10.1016/j.neuroscience.2017.01.027 28147245

[92] Evans CE Miners JS Piva G Willis CL Heard DM Kidd EJ. ACE2 activation protects against cognitive decline and reduces amyloid pathology in the Tg2576 mouse model of Alzheimer's disease. Acta Neuropathol. (2020) 139:485–502. 10.1007/s00401-019-02098-6 31982938 PMC7035243

[93] Kehoe PG Wong S Al Mulhim N Palmer LE Miners JS. Angiotensin-converting enzyme 2 is reduced in Alzheimer's disease in association with increasing amyloid- beta and tau pathology. Alzheimers Res Ther. (2016) 8:50. 10.1186/s13195-016-0217-7 27884212 PMC5123239

[94] Jiang T Tan L Gao Q Lu H Zhu XC Zhou JS. Plasma angiotensin-(1-7) is a potential biomarker for Alzheimer's disease. Curr Neurovasc Res. (2016) 13:96–9. 10.2174/1567202613666160224124739 26907614

[95] Jiang T Zhang YD Zhou JS Zhu XC Tian YY Zhao HD. Angiotensin- (1-7) is reduced and inversely correlates with tau hyperphosphorylation in animal models of Alzheimer's disease. Mol Neurobiol. (2016) 53:2489–97. 10.1007/s12035-015-9260-9 26044748

[96] Vodovar N Paquet C Mebazaa A Launay JM Hugon J Cohen-Solal A. Neprilysin, cardiovascular, and Alzheimer's diseases: the therapeutic split? Eur Heart J. (2015) 36:902–5. 10.1093/eurheartj/ehv015 25636748

[97] Barua NU Miners JS Bienemann AS Wyatt MJ Welser K Tabor AB. Convection-enhanced delivery of neprilysin: a novel amyloid-β-degrading therapeutic strategy. J Alzheimers Dis. (2012) 32:43–56. 10.3233/JAD-2012-120658 22751177

[98] Hsu FY Lin FJ Ou HT Huang SH Wang CC. Renoprotective effect of angiotensin-converting enzyme inhibitors and angiotensin II receptor blockers in diabetic patients with proteinuria. Kidney Blood Press Res. (2017) 42:358–68. 10.1159/000477946 28618426

[99] Shah SJ Stafford RS. Current trends of hypertension treatment in the United States. Am J Hypertens. (2017) 30:1008–14. 10.1093/ajh/hpx085 28531239 PMC6887988

[100] Al Khaja KAJ James H Veeramuthu S Tayem YI Sridharan K Sequeira RP. Antihypertensive prescribing pattern in older adults: implications of age and the use of dual single-pill combinations. High Blood Press Cardiovasc Prev. (2019) 26:535–44. 10.1007/s40292-019-00353-1 31797221

[101] Michaud M Balardy L Moulis G Gaudin C Peyrot C Vellas B. Pro-inflammatory cytokines, aging, and age-related diseases. J Am Med Dir Assoc. (2013) 14:877–82. 10.1016/j.jamda.2013.05.009 23792036

[102] Forlenza OV Diniz BS Talib LL Mendonça VA Ojopi EB Gattaz WF. Increased serum IL-1beta level in Alzheimer's disease and mild cognitive impairment. Dement Geriatr Cogn Disord. (2009) 28:507–12. 10.1159/000255051 19996595

[103] Dias NS Barbosa IG Kuang W Teixeira AL. Depressive disorders in the elderly and dementia: an update. Dement Neuropsychol. (2020) 14:1–6. 10.1590/1980-57642020dn14-010001 32206191 PMC7077867

[104] Diniz BS Teixeira AL Ojopi EB Talib LL Mendonça VA Gattaz WF. Higher serum sTNFR1 level predicts conversion from mild cognitive impairment to Alzheimer's disease. J Alzheimers Dis. (2010) 22:1305–11. 10.3233/JAD-2010-100921 20930310

[105] Fu Y Cheng Y Wu Y. Understanding SARS-CoV-2-mediated inflammatory responses: from mechanisms to potential therapeutic tools. Virologica Sinica. (2020) 35:266–71. 10.1007/s12250-020-00207-4 32125642 PMC7090474

[106] Mehta P McAuley DF Brown M Sanchez E Tattersall RS Manson JJ. COVID-19: consider cytokine storm syndromes and immunosuppression. Lancet. (2020) 395:1033–4. 10.1016/S0140-6736(20)30628-0 32192578 PMC7270045

[107] Wan SX Yi QJ Fan SB Lv J Zhang X Guo L Characteristics of lymphocyte subsets and cytokines inperipheral blood of 123 hospitalized patients with 2019 novel coronavirus pneumonia (NCP). MedRxiv. 10.1101/2020.02.10.20021832

[108] Dantzer R. Neuroimmune interactions: from the brain to the immune system and vice versa. Physiol Rev. (2018) 98:477–504. 10.1152/physrev.00039.2016 29351513 PMC5866360

[109] Aiba H Mochizuki M Kimura M Hojo H. Predictive value of serum interleukin-6 level in influenza virus-associated encephalopathy. Neurology. (2001) 57:295–9. 10.1212/WNL.57.2.295 11468315

[110] Ichiyama T Isumi H Ozawa H Matsubara T Morishima T Furukawa S. Cerebrospinal fluid and serum levels of cytokines and soluble tumor necrosis factor receptor in influenza virus-associated encephalopathy. Scand J Infect Dis. (2003) 35:59–61. 10.1080/0036554021000026986 12685886

[111] Jurgens HA Amancherla K Johnson RW. Influenza infection induces neuroinflammation, alters hippocampal neuron morphology, and impairs cognition in adult mice. J Neurosci. (2012) 32:3958–68. 10.1523/JNEUROSCI.6389-11.2012 22442063 PMC3353809

[112] Jurgens HA Johnson RW. Environmental enrichment attenuates hippocampal neuroinflammation and improves cognitive function during influenza infection. Brain Behav Immun. (2012) 26:1006–16. 10.1016/j.bbi.2012.05.015 22687335 PMC3454448

[113] Baig AM Khaleeq A Ali U Syeda H. Evidence of the COVID-19 virus targeting the CNS: tissue distribution, host-virus interaction, and proposed neurotropic mechanisms. ACS Chem Neurosci. (2020) 11:995–8. 10.1021/acschemneuro.0c00122 32167747

[114] Butowt R Bilinska K. SARS-CoV-2: olfaction, brain infection and the urgent need for clinical samples allowing earlier virus detection. ACS Chem Neurosci. (2020) 9:1200–3. 10.1021/acschemneuro.0c00172 32283006

[115] Elkind MS. Why now? Moving from stroke risk factors to stroke triggers. Curr Opin Neurol. (2007) 20:51–7. 10.1097/WCO.0b013e328012da75 17215689

[116] Muhammad S Haasbach E Kotchourko M Strigli A Krenz A Ridder DA. Influenza virus infection aggravates stroke outcome. Stroke. (2011) 42:783–91. 10.1161/STROKEAHA.110.596783 21293018

[117] Warren-Gash C Blackburn R Whitaker H McMenamin J Hayward AC. Laboratory-confirmed respiratory infections as triggers for acute myocardial infarction and stroke: a self-controlled case series analysis of national linked datasets from Scotland. Eur Respir J. (2018) 51:1701794. 10.1183/13993003.01794-2017 29563170 PMC5898931

[118] Wang Y Wang Y Chen Y Qin Q. Unique epidemiological and clinical features of the emerging 2019 novel coronavirus pneumonia (COVID-19) implicate special control measures. J Med Virol. (2020) 92:568–76. 10.1002/jmv.25748 32134116 PMC7228347

[119] Khoury NM Marvar PJ Gillespie CF Wingo A Schwartz A Bradley B. The renin-angiotensin pathway in post- traumatic stress disorder: angiotensin-converting enzyme inhibitors and angiotensin receptor blockers are associated with fewer traumatic stress symptoms. J Clin Psychiatry. (2012) 73:849–55. 10.4088/JCP.11m07316 22687631 PMC4087173

[120] Edmondson D Kronish IM Shaffer JA Falzon L Burg MM. Posttraumatic stress disorder and risk for coronary heart disease: a meta-analytic review. Am Heart J. (2013) 166:806–14. 10.1016/j.ahj.2013.07.031 24176435 PMC3815706

